# The Mediæval Hospitals of England

**Published:** 1910-03

**Authors:** 


**The Mediæval Hospitals of England.** By ROTHA MARY CLAY. Pp. xxii, 357. London: Methuen & Co. 1909.

In his preface to Miss Clay's book the Bishop of Bristol concludes with the words, "As a book of reference for readers and writers this treatise ought to hold a distinguished place." An expression of opinion with which we cordially agree; for, to begin at the end, the index is reliable and the tabulated list of mediæval hospitals arranged by counties as an appendix is concise and admirable, containing as it does many references to authoritative records. Bibliographical references are ungrudgingly given where they are wanted, *i.e.* at the foot of the page, in addition to a summary of leading authorities at the end of the volume. Numerous illustrations lend interest to the letterpress, though we could wish that the sources of the illustrations were in some cases made known. The author has succeeded in arranging her necessarily somewhat disjointed information in coherent order. It was no easy task to bring under a single cover the numerous worthy foundations which have held or hold the name of "hospital." The name includes every provision which mediæval generosity made for housing the aged, the infirm, the fatherless, the needy, the wayfarer, and the sick. But the task has been attempted, and the result is a book not to be set apart as a work of reference for antiquarians only, but to be read through by anyone pretending to an interest in England's past. One criticism we may venture on. Miss Clay writes of the Middle Ages as though æons separated the sixteenth from the twentieth century, yet many of the foundations she describes show marvellous little change. To those who in the twentieth century have dwelt in the foundations of the twelfth, Time's footsteps mark a shorter stride.

